# Cilostazol Upregulates Autophagy via SIRT1 Activation: Reducing Amyloid-β Peptide and APP-CTFβ Levels in Neuronal Cells

**DOI:** 10.1371/journal.pone.0134486

**Published:** 2015-08-05

**Authors:** Hye Rin Lee, Hwa Kyoung Shin, So Youn Park, Hye Young Kim, Sun Sik Bae, Won Suk Lee, Byung Yong Rhim, Ki Whan Hong, Chi Dae Kim

**Affiliations:** 1 Department of Pharmacology, School of Medicine, Pusan National University, Yangsan-si, Gyeongsangnam-do, Republic of Korea; 2 Gene & Cell Therapy Research Center for Vessel-associated Diseases, Pusan National University, Yangsan-si, Gyeongsangnam-do, Republic of Korea; 3 Medical Research Center for Ischemic Tissue Regeneration, Pusan National University, Yangsan-si, Gyeongsangnam-do, Republic of Korea; 4 Division of Meridian and Structural Medicine, School of Korean Medicine, Pusan National University, Yangsan-si, Gyeongsangnam-do, Republic of Korea; Nathan Kline Institute and New York University School of Medicine, UNITED STATES

## Abstract

Autophagy is a vital pathway for the removal of β-amyloid peptide (Aβ) and the aggregated proteins that cause Alzheimer’s disease (AD). We previously found that cilostazol induced SIRT1 expression and its activity in neuronal cells, and thus, we hypothesized that cilostazol might stimulate clearances of Aβ and C-terminal APP fragment β subunit (APP-CTFβ) by up-regulating autophagy.When N2a cells were exposed to soluble Aβ1–42, protein levels of beclin-1, autophagy-related protein5 (Atg5), and SIRT1 decreased significantly. Pretreatment with cilostazol (10–30 μM) or resveratrol (20 μM) prevented these Aβ1–42 evoked suppressions. LC3-II (a marker of mammalian autophagy) levels were significantly increased by cilostazol, and this increase was reduced by 3-methyladenine. To evoke endogenous Aβ overproduction, N2aSwe cells (N2a cells stably expressing human APP containing the Swedish mutation) were cultured in medium with or without tetracycline (Tet^+^ for 48 h and then placed in Tet^-^ condition). Aβ and APP-CTFβ expressions were increased after 12~24 h in Tet^-^ condition, and these increased expressions were significantly reduced by pretreating cilostazol. Cilostazol-induced reductions in the expressions of Aβ and APP-CTFβ were blocked by bafilomycin A1 (a blocker of autophagosome to lysosome fusion). After knockdown of the SIRT1 gene (to ~40% in SIRT1 protein), cilostazol failed to elevate the expressions of beclin-1, Atg5, and LC3-II, indicating that cilostazol increases these expressions by up-regulating SIRT1. Further, decreased cell viability induced by Aβ was prevented by cilostazol, and this inhibition was reversed by 3-methyladenine, indicating that the protective effect of cilostazol against Aβ induced neurotoxicity is, in part, ascribable to the induction of autophagy. In conclusion, cilostazol modulates autophagy by increasing the activation of SIRT1, and thereby enhances Aβ clearance and increases cell viability.

## Introduction

Alzheimer’s disease (AD) is characterized by extracellular amyloid β (Aβ)-containing plaques and intracellular neurofibrillary tangles (NFTs) consisting of aggregated phosphorylated-tau, and is accompanied by synaptic and neuronal failure and cognitive deficits [1]. Aβ and amyloid precursor protein (APP) C-terminal fragments (CTFβ) contribute to the pathology of AD and exhibit neurotoxic properties through multiple pathways [2]. Practically, failure to regulate the production and clearance of Aβ increases Aβ levels, which leads to neurotoxicity and contributes to the pathogenesis of AD [3].

Autophagy, an intracellular bulk degradation process of cellular constituents, has been reported to be highly efficient in healthy neurons and to protect them from Aβ-induced cytotoxicity [4, 5, 6], which is indicative of the neuroprotective role of autophagy against cytotoxic proteins in AD. Accordingly, defects in autophagy resulting from poor clearance of autophagosomes inside cells, is detrimental to neurons [7]. Thus, drugs that activate autophagy provide a possible alternative approach to the degradations of Aβ and APP-CTF in AD.

Evidence obtained from a mouse model indicates that calorie restriction attenuates β-amyloid neuropathology in AD [8, 9]. Qin et al. [10] described a role for SIRT1 activation by calorie restriction in the modulation of β-amyloid neuropathology in the AD brain. In one study, SIRT1 was shown to activate autophagy by deacetylating several essential components of the autophagy machinery, such as, autophagy-related genes like Atg5, Atg7, and Atg8 [11]. Beclin-1 plays an initiating role as an essential component of the autophagic pathway [12, 13]. Furthermore, three more components of the autophagy pathway, namely, Atg5, beclin-1, and Ulk1, have been shown to be involved in the degradations of Aβ and APP-CTF [14]. Mizushima and Yoshimori [15] showed microtubule-associated protein light chain 3 (LC3), which is localized at autophagosome membranes, is involved in the monitoring of autophagy.

Cilostazol increases intracellular cyclic AMP (cAMP) levels by inhibiting type III phosphodiesterase. A clinical trial reported a pilot study on 10 patients with moderate Alzheimer’s disease in a clinical setting where combination therapy of donepezil with cilostazol significantly improved the Mini-Mental State Exam (MMSE) score and maintained the current status unchanged until the end of the follow-up period in human patients with AD [16]. In addition to such effects, Park et al. [17] have reported cilostazol reduces intracellular Aβ and phosphorylated tau levels in N2a cells stably expressing human APP Swedish mutation (N2aSwe cells), and in-line with these results, cilostazol significantly improved brain function such as spatial learning and memory in an experimental model of Alzheimer’s disease. Most recently, cilostazol was documented to be effective in ameliorating cognitive decline in patients with AD with cerebrovascular diseases [18] and mild cognitive impairment [19]. In addition, we recently reported cilostazol-stimulated CK2/SIRT1 activation suppressed tau acetylation and phosphorylation by inhibiting the activations of P300 and GSK3β, and decreasing Aβ expression in N2aSwe cells [20].

Given (1) autophagy is a major cellular pathway for the removal of β and aggregated proteins, and (2) cilostazol stimulates the expression and activity of SIRT1; we hypothesized that the therapeutic use of cilostazol to enhance the autophagy pathway might provide an attractive pharmacological direction for decreasing intracellular Aβ and APP-CTFβ levels in AD. Thus, in the present study, we investigated whether cilostazol protects N2a cells from Aβ-induced neurotoxicity by up-regulating the autophagy machinery and its associated proteins (beclin-1, Atg5, and LC3-II). In addition, we sought to elucidate the mechanism whereby cilostazol inhibits Aβ-induced decreased autophagy in N2aSwe cells.

## Results

### Time-dependent decreases in beclin-1, Atg5, and SIRT1 expressions in N2a cells in response to exogenous Aβ 1–42 and cilostazol effects

**Fig 1:**
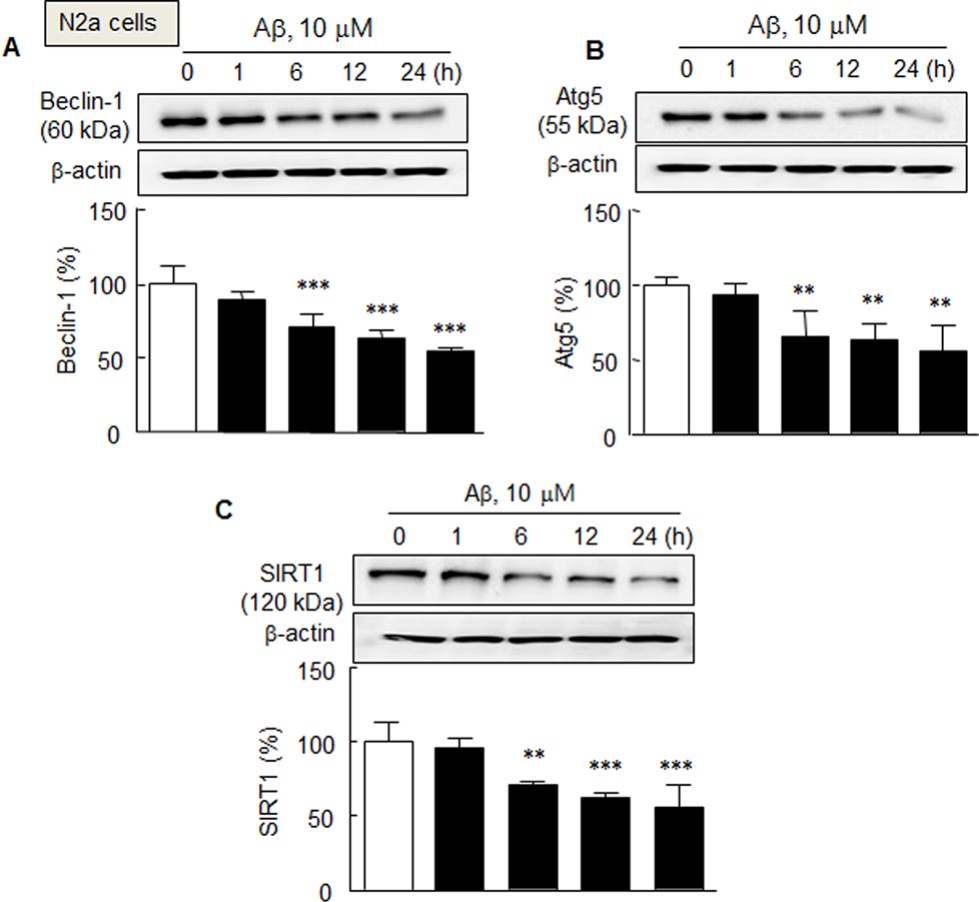
Time-dependent decreases in beclin-1 (A), Atg5 (B), and SIRT1 (C) protein expression in N2a cells exposed to exogenous Aβ1–42 (10μ μM). Means ± SDs are expressed as percentages of zero time values (N = 4). **P < 0.01, ***P < 0.001 vs. Zero time.

To determine whether Aβ1–42 affects beclin-1, Atg5, and SIRT1 levels, we examined their protein expressions in N2a cells. Exposure to Aβ1–42 (10 μM) significantly decreased levels of beclin-1 (*F*_4,15_ = 28.15, *P* < 0.0001), Atg5 (*F*_4,15_ = 10.16, *P* < 0.0003), and SIRT1 (*F*_4,15_ = 16.94, *P* < 0.0001) in a time (1, 6, 12 and 24 h)-dependent manner (Fig 1A, 1B and 1C).

**Fig 2:**
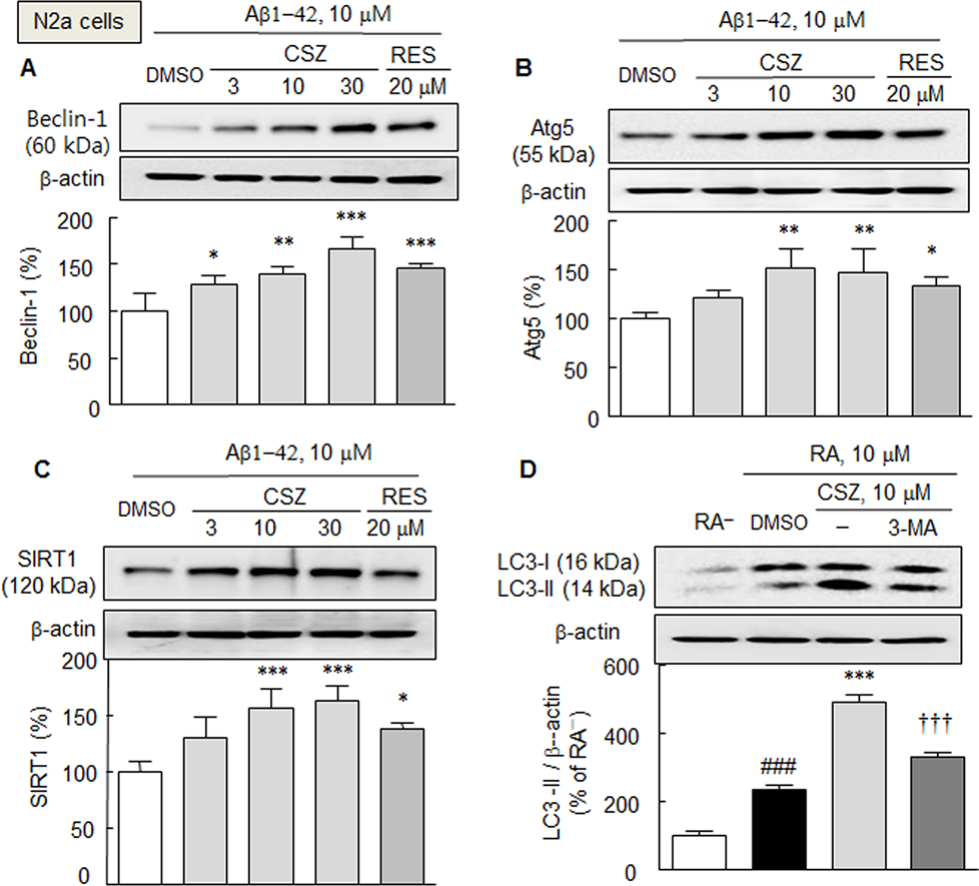
Increases in the expressions of beclin-1 (A), Atg5 (B), and SIRT1 protein (C) by cilostazol (CSZ, 3–30 μM; incubation for 3 h) and resveratrol (RES, 20 μM) in the presence of exogenous Aβ1–42 (10 μM) in N2a cells. D. Enhancement of LC3-II levels in the culture media containing 10 μM retinoic acid by cilostazol (10 μM), and its blockade by 3-methyladenine (3-MA, 2.5 mM). Means ± SDs are expressed as percentages of DMSO (vehicle) or absence of retinoic acid (RA^-^) (N = 4). ^###^ *P* < 0.001, RA^−^; *P < 0.05, **P < 0.01, ***P < 0.001 vs. DMSO; ^†††^ *P* < 0.001 vs. 10 μM cilostazol.

However, when cells were pretreated with cilostazol (3, 10, or 30 μM) or resveratrol (20 μM) for 3 h prior to exposure to exogenous Aβ1–42 (10 μM) in medium, beclin-1 expression reduction by Aβ1–42 was significantly prevented and rather reversed by cilostazol (by 140.0 ± 7.3% and 165.8 ± 13.2% at 10 and 30 μM, respectively) (*F*_3,12_ = 16.48, *P* < 0.0001). Similarly, pretreatment with cilostazol (3, 10 and 30 μM) also significantly reversed Aβ1-42-induced reductions in the expressions of Atg5 (*F*_3,12_ = 6.39, *P* < 0.0033) and SIRT1 (*F*_3,12_ = 11.86, *P* < 0.0002). Similarly, resveratrol (20 μM) also prevented Aβ1-42-induced reductions of beclin-1(by 145.2 ± 5.4%, *P* < 0.001), Atg5 (by 122.6 ± 20.3%, *P* < 0.05), and SIRT1 protein (by 120.9 ± 10.2%, *P* < 0.05), respectively (Fig 2A, 2B and 2C).

It has been reported that the transient expression of wild-type SIRT1 stimulates the conversion of LC3-I to LC3-II in HCT116 cells [11]. To investigate the effect of cilostazol on LC3-II expression, a model of retinoic acid-induced neuronal differentiation was used. As shown in Fig 2D, LC3-II levels in N2a cells were elevated by 238 ± 11.0% (*P* < 0.001) in culture media containing 10 μM retinoic acid as compared with the non-retinoic acid control, and interestingly, cilostazol (10 μM) increased LC3-II levels by 491.3 ± 21.9% (*P* < 0.001). Furthermore, this effect of cilostazol was significantly blocked by 3-methyladenine (2.5 mM; an inhibitor of autophagy), indicating that cilostazol enhanced autophagosome formation.

### Increases in Full-length APP, Aβ and CTFβ levels in N2aSwe cells and the inhibitory effect of cilostazol

**Fig 3:**
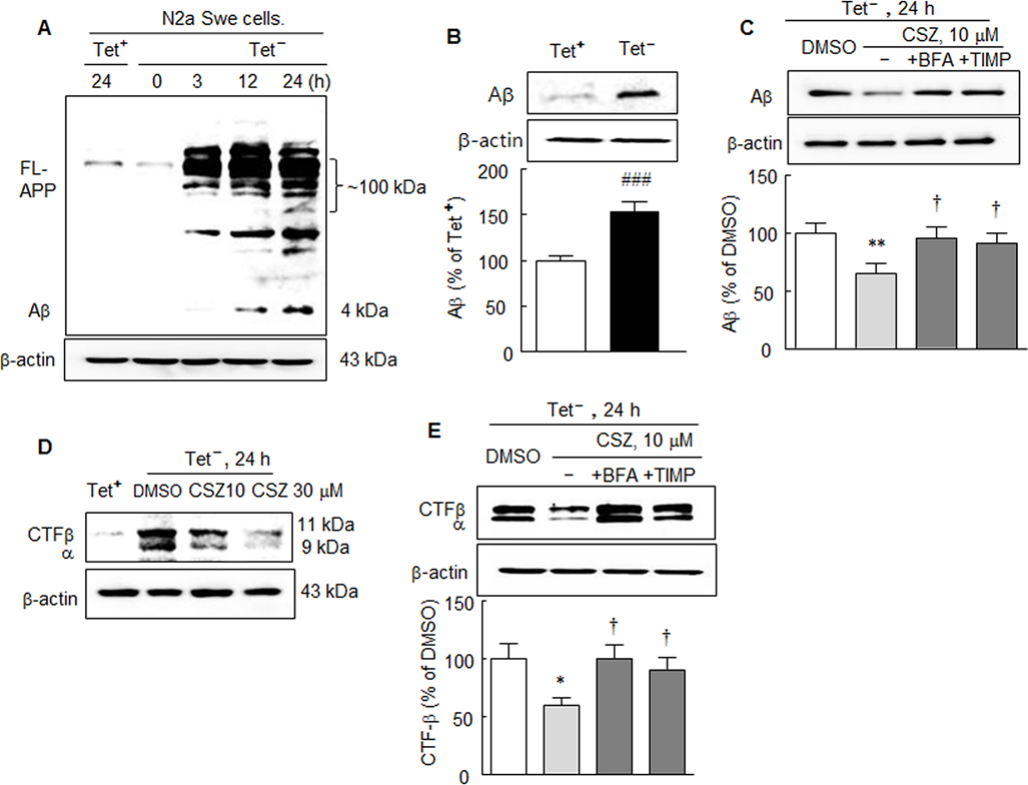
A. Time-dependent increases in the expressions of full-length APP (FL-APP) and Aβ in N2aSwe cells determined using anti-Aβ (6E10) antibody. To evoke endogenous Aβ overproduction, cells were exposed to medium containing 1 μg/ml of tetracycline (Tet^+^) for 48 h and then switched to tetracycline-free medium (Tet^-^) for 3, 12, and 24 h. B. Aβ accumulation after culturing cells in Tet^-^ condition for 24 h. C. Cilostazol-induced suppression of Aβ expression induced by Tet^-^ condition, and prevention of this inhibition by bafilomycin A1 (BFA, 100 nM) and TIMP-1 (10 μM), respectively. D. Effect of cilostazol on the increased CTFβ (11 kDa) and CTFα (9 kDa) levels cultured in Tet^-^ condition (Western blot using rabbit polyclonal CTFβ (751–770) antibody). The Western blots shown are representative of four independent experiments that yielded similar results. E. Prevention by bafilomycin A1 (BFA, 100 nM) and TIMP-1 (10 μM) of cilostazol-induced decreases in the CTFβ expressions. Results are the means ± SDs of percentages (N = 4). ^###^ *P* < 0.001 vs. Tet^+^; **P < 0.01, ***P < 0.001 vs. DMSO; ^†^ *P* < 0.05 vs. cilostazol (CSZ, 10 μM) alone.

To investigate endogenous accumulations of full-length APP and Aβ under pathological situations simulating those in the AD brain, we used mouse neuroblastoma cells stably expressing human APP Swedish mutation (N2aSwe cells). These cells were exposed to Tet^+^ and Tet^-^ conditions, as previously described by Anekonda et al. [21]. Briefly, cells were exposed to medium containing 1 μg/ml of tetracycline (Tet^+^) for 48 h, and then removed to tetracycline-free (Tet^-^) conditions for 3, 12, or 24 h to induce endogenous Aβ overproduction. As shown in Fig 3A, in Tet^+^ condition, cells showed thin density, but in the Tet^-^ condition, N2aSwe cells exhibited time-dependent increases in full-length APP (~100 kDa) at between 3 and 24 h (using anti-Aβ (6E10) antibody). Accordingly, the accumulation of Aβ (4 kDa) increased from 12 ~ 24 h under the Tet^-^ condition by 165.4 ± 15.0% (*P* < 0.001) (Fig 3B). This increase in Aβ level was significantly reduced by 65.1 ± 9.0% (*P* < 0.01) under pretreatment with 10 μM of cilostazol, and this reduction by cilostazol was prevented by co-treating cilostazol with bafilomycin A1 (100 nM; a blocker of autophagosome to lysosome fusion) [22] by 95.6 ± 9.1%, *P* < 0.05 and with TIMP-1 (10 μM, by 91.0 ± 8.7%, *P* < 0.05), an ADAM10 inhibitor [23] (Fig 3C).

In line with these results, β and α subunit levels of APP-CTF protein (as determined using rabbit polyclonal CTFβ (751–770) antibody) were markedly increased upon exposure to the Tet^-^ condition. These increased levels of both subunits were similarly decreased by cilostazol (10 or 30 μM) treatment in a concentration-dependent manner (Fig 3D), and this increase was significantly reduced by 59.3 ± 6.9% (*P* < 0.05) after treatment with cilostazol (10 μM). Furthermore, this decrease in CTFβ expression elicited by cilostazol was significantly inhibited by bafilomycin A1 (by 99.6 ± 12.0%, *P* < 0.05) and by TIMP-1 (10 μM, by 84.0 ± 10.0%, *P* < 0.05) (Fig 3E).

### Beclin-1, Atg5 and SIRT1 reductions in N2aSwe cells in response to endogenously overproduced Aβ and inhibition of these reductions by cilostazol

**Fig 4:**
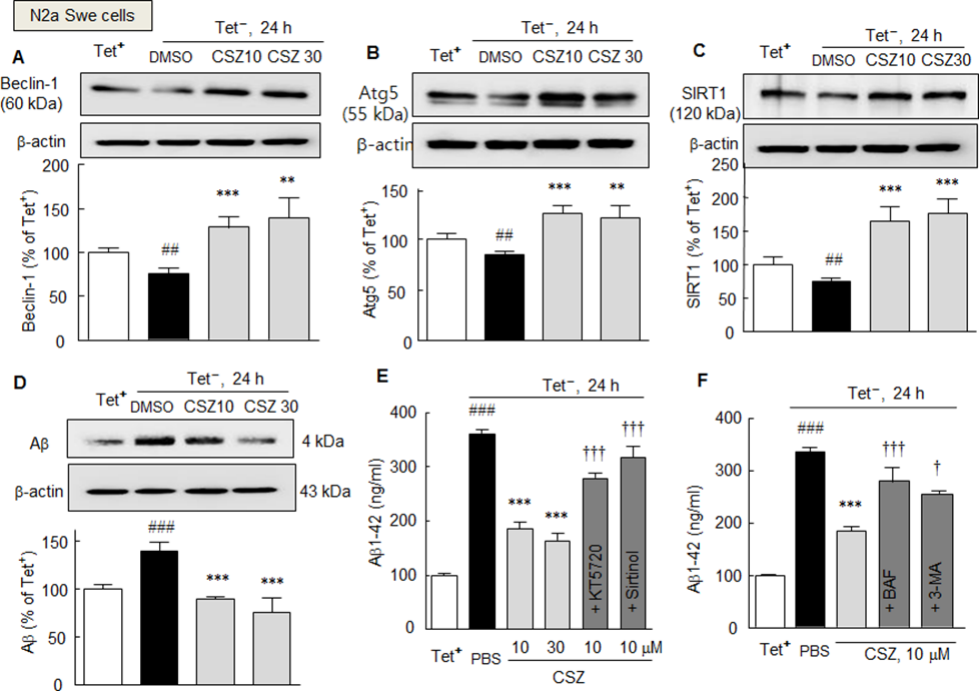
A—C. Inhibition of Tet^-^ condition-induced reductions in beclin-1 (A), Atg5 (B) and SIRT1 expressions (C) by cilostazol (10 or 30 μM) in N2aSwe cells. D. Inhibition of Tet^-^ condition-induced endogenous increases in Aβ level by cilostazol as determined by Western blotting (D) and of intracellular Aβ accumulation as determined by ELISA (E, F). The inhibitory effects of cilostazol were blocked by KT5720 (1 μM), sirtinol (20 μM) (E), bafilomycin A1 (BAF, 100 ng/ml), or 3-methyladenine (3-MA, 2.5 mM) (F). Results are presented as means ± SDs (N = 4–5). ^##^ *P* < 0.01, ^###^ *P* < 0.001 vs. Tet^+^ condition (as control), **P < 0.01 ***P < 0.001 vs. DMSO; ^†^ *P* < 0.05, ^†††^ *P* < 0.001 vs. cilostazol alone (CSZ, 10 μM). PBS, phosphate-buffered saline.

We further confirmed cilostazol significantly inhibited endogenously overproduced Aβin N2aSwe cells induced by using the model of Tet^+^ and Tet^-^ conditions. As shown in Fig 4A, 4B and 4C, the protein expressions of beclin-1, Atg5, and SIRT1 were significantly reduced by 76.1 ± 6.8%, 85.3 ± 3.2% and 76.8 ± 6.4%, respectively (each, *P* < 0.01) when N2aSwe cells were exposed to Tet^-^. Interestingly, by pretreatment with cilostazol (10 or 30 μM), these attenuated levels of SIRT1, beclin-1, and Atg5 were largely overexpressed (10 μM cilostazol: by 155.1 ± 18.1%, 136.6 ± 11.7%, and 125.7 ± 8.0%, respectively). Intriguingly, increased Aβ level under Tet^-^ condition (by 139.5 ± 9.8%, *P* < 0.001) was significantly inhibited by cilostazol pretreatment (10 or 30 μM) to 89.4 ± 2.4 (*P* < 0.001) and 75.5 ± 16.0% (*P* < 0.001), respectively (Fig 4D). These results were further evaluated by measuring Aβ levels by ELISA. When N2aSwe cells were exposed to Tet^-^ condition, intracellular Aβ1–42 levels significantly increased by 362.2 ± 7.7 ng/ml (*P* < 0.001), and this increase was markedly reduced to 186.4 ± 10.9 ng/ml (*P* < 0.001) and 165.9 ± 10.9 ng/ml (*P* < 0.001) by pretreating cilostazol at 10 and 30 μM, respectively. Furthermore, this cilostazol-induced inhibition was significantly blocked by pretreatment with KT5720 (1 μM, a cAMP-dependent protein kinase inhibitor) or sirtinol (20 μM, a SIRT1 inhibitor) (Fig 4E). In addition, cilostazol-induced inhibition of Aβ1–42 level was also blocked by bafilomycin A1 (100 nM, *P* < 0.001) or 3-methyladenine (2.5 mM, *P* < 0.05), respectively (Fig 4F). Overall, these results indicate cilostazol stimulates beclin-1, Atg5 and SIRT1 expression even under Aβ-enriched conditions, and that cilostazol inhibits intracellular Aβ accumulation by elevating autophagy.

### Failure of cilostazol to elevate autophagy in SIRT1-gene silenced cells

**Fig 5:**
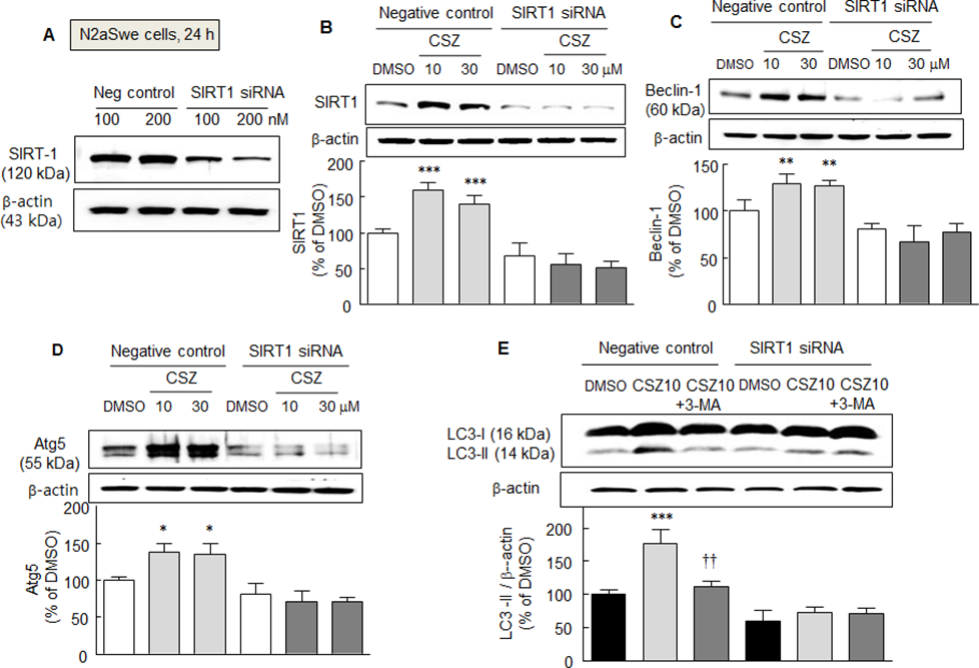
A. Analysis of the effects of SIRT1-knockdown in N2aSwe cells. Proteins (30 μg) from a negative control and SIRT1- knockdown samples were loaded onto 10 ~ 15% SDS-polyacrylamide gels. In N2aSwe cells transfected with 200 nM of SIRT1 siRNA, SIRT1 protein levels were at ~ 40% of the level in negative controls (A). Cilostazol failed to elevate the expressions of SIRT1 (B), beclin1 (C), Atg5 (D), and (E) LC3 in SIRT1-siRNA treated N2a cells, as contrasted to the levels of all four in the negative control cells. Results are the means ± SDs of 4 experiments. *P < 0.05, **P < 0.01, ***P < 0.001 vs. DMSO. ^††^ *P* < 0.01 vs. cilostazol alone (CSZ, 10 μM).

To confirm cilostazol-induced increases in beclin-1, Atg5, and LC3-II levels are mediated via SIRT1 activation, N2aSwe cells were transfected with SIRT1 siRNA or scrambled siRNA duplex (negative control) (both at 100 or 200 nM). In N2aSwe cells subjected to SIRT1-gene silencing, SIRT1 protein expression was reduced to ~ 40% by 200 nM of SIRT1 siRNA oligonucleotide (Fig 5A). In SIRT1- silenced cells, cilostazol failed to elevate SIRT1 protein expression, whereas in cells transfected with scrambled siRNA duplex (negative control), cilostazol at 10 or 30 μM significantly elevated the expression of SIRT1 to 159.2 ± 11.2% and 139.8 ± 13.2%, respectively (Fig 5B). Similarly, beclin-1, Atg5, and LC3-II levels were not increased by cilostazol (10 or 30 μM) in cells transfected with SIRT1 siRNA, whereas levels of these proteins were increased in cells transfected with scrambled siRNA duplex (negative control) (Fig 5C, 5D and 5E). These results show cilostazol increases the expressions of beclin-1, Atg5, and LC3-II by up-regulating SIRT1.

### Immunoprecipitation and Immunofluorescence studies

**Fig 6:**
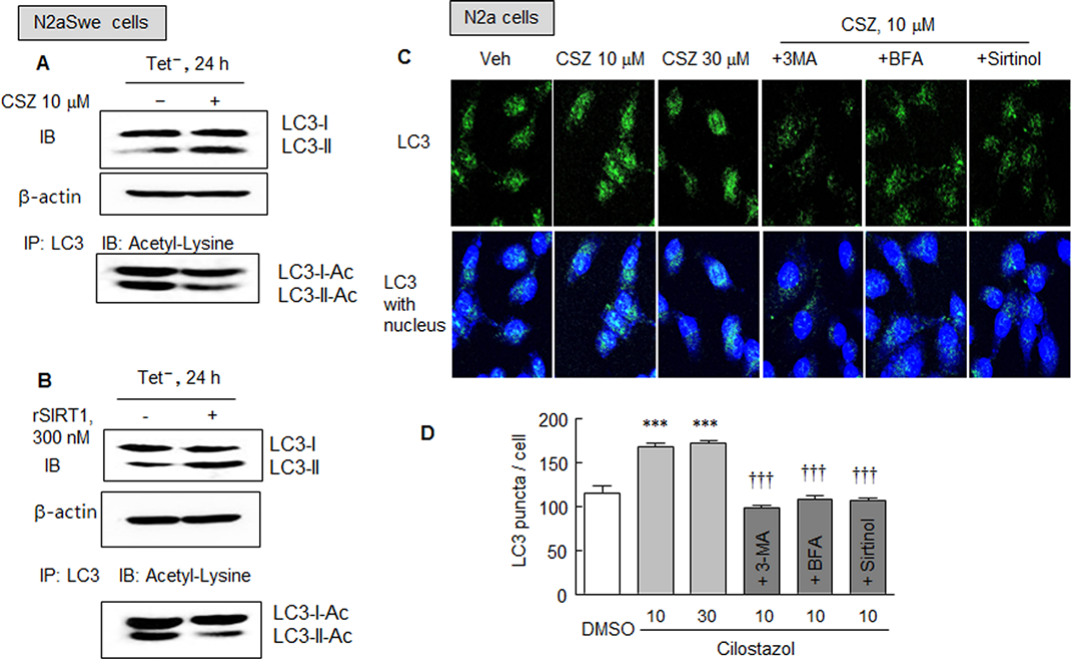
A and D. Immunoprecipitation analysis. Whole cell lysates were obtained from N2aSwe cell lysates that were cultured in Tet^-^ condition for 24 h with or without cilostazol (10 μM, A) or rSIRT1 (recombinant SIRT1, 300 nM; B). Upper panels (A and B): the effects of cilostazol and rSIRT1 on LC3-II expression were confirmed. Lower panels (A and B): cell lysates were immunoprecipitated with LC-3 antibody and then immunoblotted for acetylated LC3 LC3-1/II-Ac) using an anti-acetyl lysine antibody. The blot shown is representative of three experiments that produced similar results. C. Immunofluorescent assay of LC3 puncta in N2a cells treated with or without cilostazol (10 and 30 μM) after being pretreated with 3-methyladenine (2.5 mM), bafilomycin A1 (100 nM) or sirtinol (20 μM). D. Quantitative analysis was performed by counting numbers of LC3 puncta/cell. Results are the means ± SDs of 4 experiments. ***P < 0.001 vs. DMSO. ^†††^ *P* < 0.001 vs. cilostazol (10 μM).

To examine the effect of SIRT1 on autophagy components, we assessed and compared the deacetylations of LC3 by cilostazol or recombinant SIRT1 (rSIRT1) in the N2aSwe cells. Neuronal cells were exposed to Tet^-^ for 24 h in the absence and presence of 10 μM of cilostazol or 300 nM of rSIRT1, and then one part of each whole cell lysates was immunoblotted for LC3. The other portions were immunoprecipitated with LC-3 antibody and immunoblotted for acetylated LC3 using an anti-acetyl lysine antibody. As shown in Fig 6A and 6B, LC3-II levels were markedly elevated by treatment with cilostazol or rSIRT1. However, after immunoprecipitating LC-3, the immunoblotted band intensity of acetylated LC3-II was markedly reduced (by ~30%) in samples treated with cilostazol or rSIRT1.

In addition, the effect of cilostazol was further examined with respect to autophagy initiation by assaying LC3 puncta in N2a cells treated with or without cilostazol (10 or 30 μM). Fluorescent puncta were significantly increased by cilostazol at 10 and 30 μM (both *P* < 0.001), but these increases were significantly blocked by 3-methyladenine (2.5 mM, *P* < 0.001), bafilomycin A1 (100 nM, *P* < 0.001), or sirtinol (20 μM, *P* < 0.001). Overall, these results show that like rSIRT1, cilostazol stimulates the conversion of LC3-I to LC3-II, which is suggestive of increased autophagosome formation.

### Cell viability enhancement by cilostazol

**Fig 7:**
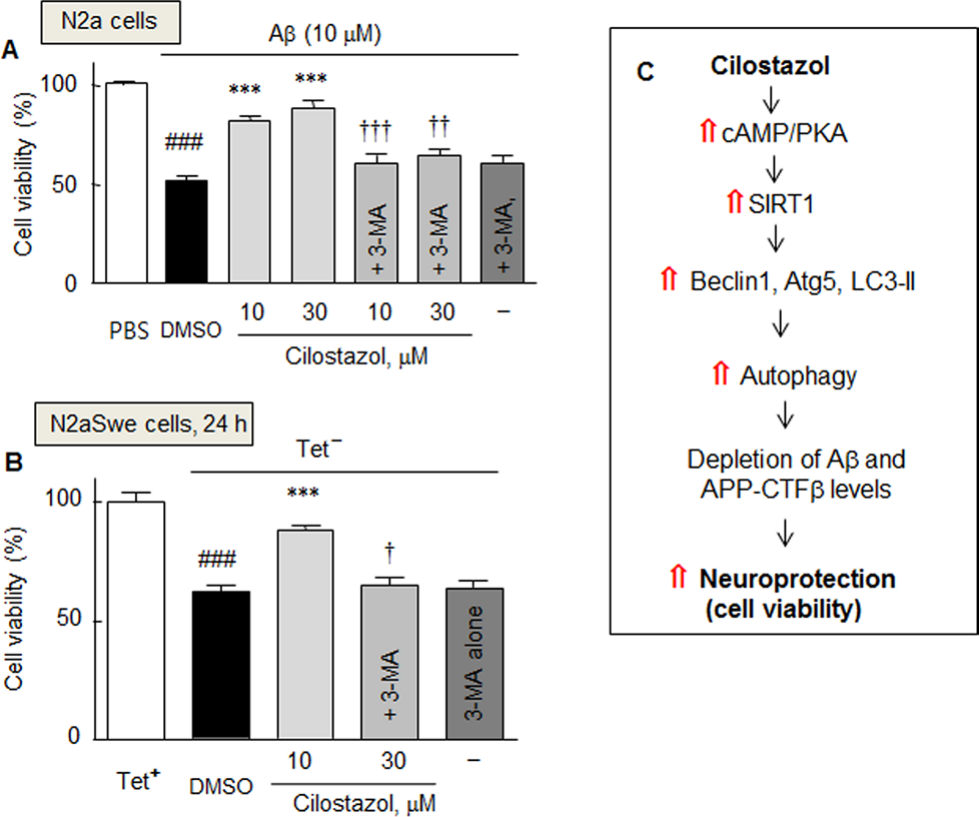
Effect of cilostazol on Aβ-induced cytotoxicity. Decrease in cell viability in response to exogenous Aβ1–42 in N2a cells (A) and to endogenously overproduced Aβ in the N2aSwe cells (B), and the recovery by cilostazol in the absence and presence of 3-methyladenine (3-MA, 2.5 mM). Results are the means ± SDs of three experiments. ^###^ *P* < 0.001 vs. PBS (A) and Tet^-^ condition (B); ***P < 0.001 vs. DMSO; ^†^ *P* < 0.05, ^††^ *P* < 0.01, ^†††^ *P* < 0.001 vs. 10 or 30 μM cilostazol alone. PBS, phosphate-buffered saline. C. Hypothetical model: Neuroprotective effect of cilostazol against Aβ-induced neurotoxicity is ascribable to the increased induction of autophagy by increasing the cAMP/PKA coupled SIRT1 activation, thereby enhances Aβ and CTFβclearance and increases cell viability.

The cytotoxic effects of exogenously applied Aβ1–42 in N2a cells and of endogenously released Aβ in N2aSwe cells were assessed using an MTT assay. Exposure of N2a cells to 10 μM of Aβ1–42 for 24 h resulted in a significant decline in cell viability by 51.6 ± 2.7% (*P* < 0.001). The decreased viability induced by Aβ1–42 was largely recovered by 10 or 30 μM of cilostazol to 81.7 ± 2.6% (*P* < 0.001) and 88.0 ± 3.7% (*P* < 0.001), respectively. Furthermore, this effect of cilostazol was significantly blocked by pretreating cells with 3-methyladenine (2.5 mM, a chemical inhibitor of autophagy) (Fig 7A).

In addition, under exposure to endogenously released Aβ, cell viability significantly decreased to 62.4 ± 2.0% (*P* < 0.001), and this was prevented by pretreating with 10 μM of cilostazol to 88.1 ± 1.8% (*P* < 0.001). Furthermore, this effect of cilostazol was blocked by pretreating cells with 3-methyladenine (2.5 mM) to 64.5 ± 3.5% (*P* < 0.05), indicating cilostazol failed to protect neurons from Aβ toxicity when autophagy was blocked (Fig 7B). These results suggest cilostazol protects against Aβ-induced neurotoxicity by inducing autophagy. Fig 7C provides a hypothetical model for the cilostazol-coupled cAMP/PKA-mediated induction of autophagy upregulation, including the expressional up-regulations of beclin-1, Atg5, and LC3-II via SIRT1 activation, which reduces intracellular Aβ and APP-CTFβ and increases cell viability.

## Discussion

The present study demonstrates both applied Aβ1–42 and endogenously generated Aβ from activated N2aSwe cells decrease the expressions of beclin-1, Atg5, and SIRT1 at the protein level, and that these suppressions are prevented by cilostazol pretreatment. Interestingly, cilostazol-stimulated upregulations of SIRT1-associated beclin-1, Atg5, and LC3-II reduced the accumulations of Aβ and APP-CTF β in N2aSwe cells. Furthermore, the decreased viability induced by Aβ was largely prevented by cilostazol, and this prevention was blocked by 3-methyladenine. These results suggest cilostazol protects against Aβ- induced neurotoxicity by enhancing the induction of autophagy. The present results also reveal that increases in Aβ and CTFβ expressions induced by Tet^+^ and Tet^-^ procedures are attenuated by cilostazol. Previous study [24] also showed activated N2aSwe cells by depletion of FBS (1%) induced large accumulations of full-length APP (100 kDa), which peaked at 12 h post activation. These accumulations were significantly and concentration-dependently diminished by pretreatment with cilostazol (3 ~ 30 μM).

Many authors have reported autophagy induces the degradation of aggregated proteins that cause AD, and that dysfunction of the autophagy-lysosome system contributes to Aβaccumulation and to the formation of tau oligomers [25–27]. Therefore, pharmacological regulation of autophagy-lysosome protein degradation is emerging as an important strategy for the treatment of AD.

Intriguingly, Lee et al. [11] emphasized SIRT1 deacetylase is an important regulator of autophagy: they showed that transiently augmenting SIRT1 activity is sufficient to activate autophagy, whereas SIRT1^-/-^ mouse embryonic fibroblasts did not fully activate autophagy under starvation conditions. In addition, the level of beclin-1, a protein that plays a key role in autophagy, was reported to be diminished in the affected brain regions of AD patients, although other studies failed to observe this effect [28–30].

To explore relationships between the effects of Aβ and the expressions of molecular components, such as, beclin-1, Atg5, and LC3-II, of the machinery of autophagy under pathological situations, we evoked endogenous Aβ overproduction in N2aSwe cells. N2aSwe cells were exposed to Tet^+^ for 48 h, and then placed to Tet^-^ condition instead of using serum-depleted culture medium, because we observed that serum depletion *per se* stimulates the upregulations of beclin-1 and Atg5 in a manner similar to starvation conditions. When N2aSwe cells were exposed to Tet^+^/Tet^-^, they exhibited time-dependent increases in Aβ and APP-CTFβ levels. The present results also showed elevated intracellular Aβ1–42 levels were markedly inhibited by cilostazol and that this inhibition was blocked by KT5720 and sirtinol, indicating that cAMP-dependent protein kinase and SIRT1 underlie the action mechanism of cilostazol, as has been previously reported by Lee et al. [20].

Our observations of increases in beclin-1, Atg5, and SIRT1 levels by cilostazol (10 or 30 μM) are considered to be related to reduced accumulations of Aβ and APP-CTFβ, which were induced by Tet^-^ in the presence of cilostazol, because the cilostazol-induced suppressions of APP-CTFβ expression and intracellular Aβlevels were significantly blocked by bafilomycin A1 (a blocker of autophagosome to lysosome fusion) [22] or 3-methyladenine (an inhibitor of autophagy) [31]. In light of the facts that cilostazol-induced decreases in CTFβ and Aβ accumulation were significantly inhibited by bafilomycin A1 or TIMP-1 (an ADAM10 inhibitor) [23], the inhibition of CTFβ and Aβ accumulation by cilostazol is ascribed to increased autophagic clearance and to reduced production of Aβ through ADAM 10 (α-secretase) activation [24].

It was also reported some time ago LC3 is necessary for the formation of autophagosomes and that it localizes to autophagosome membranes [32]. The conversion of LC3-I to LC3-II via proteolytic cleavage is a hallmark of mammalian autophagy: the amount of LC3-II and LC3-II/LC3-I ratio are closely related to autophagosome formation [33]. Thus, to evaluate the level of autophagy in a more specific way, we assessed levels of autophagy protein LC3, a marker of mammalian autophagy. Cilostazol markedly increased LC3-II levels in N2a cells, and this increase was significantly blocked by 3-methyladenine, indicating that the autophagy pathway is up-regulated by cilostazol. Interestingly, after immunoprecipitating LC-3, the acetylated LC3-II band was markedly decreased (by ~30%) in cells pretreated with cilostazol or rSIRT1. Moreover, numbers of immunofluorescent puncta were significantly increased by cilostazol, and this increase was blocked by 3-methyladenine, bafilomycin A1, or sirtinol, respectively. These results strongly suggest that like rSIRT1, cilostazol stimulates the conversion of LC3-I to LC3-II, which is indicative of increased autophagosome formation.

To confirm that the cilostazol-induced elevations of beclin-1 and Atg5 were mediated by the activation of SIRT1, N2aSwe cells were transfected with SIRT1 siRNA. After silencing the SIRT1 gene, the expressions of beclin-1, Atg5, LC3-II, and SIRT1 were not induced by cilostazol, whereas negative control cells (transfected with scrambled siRNA duplex) were obviously responsive to cilostazol. These observations indicate that increases in beclin-1, Atg5, and LC3-II levels were evoked by cilostazol via SIRT1 activation.

These postulations are strongly supported by the previous reports, in that cilostazol rescued HT22 apoptosis induced by Aβ toxicity by downregulating phosphorylated p53 (Ser 15), Bax, and caspase-3 levels and upregulating Bcl-2 levels, and protected against the suppression of neurite elongation by Aβ [34]. Moreover, cilostazol suppressed the accumulations of Aβ by increasing the expression of ADAM10 and α-secretase activity via the upregulation of cAMP-dependent protein kinase-linked SIRT1 expression in activated N2aSwe mutant cells [24]. Based on these reports and the present study, it is considered that cilostazol prevents Aβ-induced cell viability reduction. A question arises as to how cilostazol clears APPswe metabolites released from N2aSwe cells, since clearance of metabolites generated from APPswe cells may mechanistically differ from soluble β-amyloid as suggested by Haass et al. [35]. Further study is required to define the autophagic assessment of metabolites from APPswe cells by cilostazol.

Taking these results and those regarding the pharmacological inhibition and gene silencing of SIRT1, cilostazol appears to protect neuronal cells from Aβ-induced neurotoxicity by up-regulating the autophagy machinery, as demonstrated by the up-regulations of beclin-1, Atg5, and LC3-II, via activating SIRT1 expression and decreasing Aβ peptide production, and thereby improves cell viability (Fig 7C).

## Materials and Methods

### Cell culture

Mouse neuroblastoma N2a wild-type cells and N2aSwe cells were kindly donated by Dr. Takeshi Iwatsubo (Department of Neuropathology and Neuroscience, Graduate School of Pharmaceutical Sciences, The University of Tokyo) [36]. These cells were cultured in media containing 45% Dulbecco’s Modified Eagle’s Medium (DMEM, Gibco, Carlsbad, CA), 55% Opti-MEM (Gibco), supplemented with 10% fetal bovine serum (Hyclone, Logan, UT), 100 units/ml penicillin, 100 μg/ml streptomycin, 1% glutamine, and 0.09% Hygromysin B (Sigma-Aldrich, St. Louis, MO) in a humidified 5% CO_2_/ 95% air atmosphere at 37°C. To evoke endogenous Aβ overproduction, N2a and N2aSwe cells were cultured under the above condition in the presence of 1 μg/ml of tetracycline (Tet^+^, which was used as a control) for 48 h and then placed in tetracycline-free condition (Tet^-^) and cultured for 3, 12, and 24 h, respectively. When treatment with either cilostazol or resveratrol was required, cells were pretreated with these drugs for 3 h in Tet^+^, and then switched to Tet^-^ condition containing the same drugs and cultured for the indicated times.

### Western blotting

For Western blot analyses, cells were scraped, and lysed in buffer A containing 10 mM HEPES, 10 mM NaCl, 1.5 mM MgCl2, 0.25% Tween 20, 1mM dithiothreitol, 100 mg/ml phenylmethylsulfonyl fluoride, 1 mg/ml leupeptin, and 15 mg/ml aprotinin. Thirty μg of total protein from each sample was then loaded onto 10~15% SDS-polyacrylamide gels, because all sorts of proteins measured in this experiment have a diversity of molecular weights (from 4 ~ 120 kDa). Separated proteins were transferred to nitrocellulose membranes (GE Healthcare Life Sciences, Piscataway, NJ, USA), which were blocked with 5% skim milk (at 4°C overnight), and incubated with antibodies against SIRT1 (Santa Cruz, CA), anti-Aβ (6E10) (Covance, Emeryville, CA), and rabbit polyclonal CTFβ (751–770) (Calbiochem, La Jolla CA. Antibodies against beclin-1, Atg5, and LC3 (rabbit monoclonal LC3A/B, 1:1000) were obtained from Cell Signaling Technology (Danvers, MA). Membranes were reprobed with an anti-β-actin antibody (MP Biomedicals, LLC, Aurora, OH, USA) as an internal control.

### Measurement of Aβ levels by ELISA

Cell lysates from cilostazol-treated and untreated cells were collected, and Aβ1–42 levels were determined using ELISA kit Aβ1–42 (FIVEphoton Biochemicals, San Diego, CA). Optical densities were read at 450 nm using a plate reader, and Aβ1–42 concentrations were determined using standard curves. All readings taken fell within the linear range of the assay.

### Immunofluorescence experiments

For immunofluorescence studies, N2a cells were fixed in 4% (w/v) paraformaldehyde for 30 min at room temperature, permeabilized with PBS containing 3% bovine serum albumin and 0.1% (v/v) Triton X-100 for 30 min, and then incubated for 1 h at room temperature under constant shaking with antibody against LC3 (rabbit monoclonal LC3A/B antibody; dilution 1:1,000; Cell Signaling). After several washes with PBS, cells were incubated for 1 h with secondary antibody conjugated to Alexa Fluor 488 and 594 (Invitrogen, Carlsbad, CA) at room temperature, washed again with PBS, labeled with DAPI, and mounted in Fluoprep (Bio-Merieux, Craponne-Pays, France). All fluorescent images were acquired at ×100 using an Axiovert 200 (Carl Zeiss, Jena, Germany) fluorescence microscope.

### SiRNA SIRT1 transfection assays

N2aSwe cells were grown on 6-well plates (1×10 ^5^ cells/well), placed in 60-mm dishes (1–2 × 10^6^ cells/well) coated with poly-D-lysine in Neurobasal medium containing B-27, and then transfected for 8 h with 100–200 nM of siRNA (small interfering RNA). SIRT1 siRNA oligonucleotides (GenBank accession No. NM_003120.1) were synthesized by Bioneer (Daejeon, Korea). A scrambled siRNA duplex was used as the control oligonucleotide. siRNA sequences against SIRT1 were; ACGAUGACAGAACGUCACA (sense), and UGUGACGUUCUGUCAUCGU (antisense).

### Immunoprecipitation

For the immunoprecipitation assay, N2aSwe cells were lysed with lysis buffer containing 50 mM HEPES-OH pH 7.5, 120 mM NaCl, 1 mM EDTA, 10 mM pyrophosphate, 10 mM glycerophosphate, 50 mM NaF, 1 mM PMSF, 1.5 mM Na_3_VO_4_, 0.3% CHAPS, and protease inhibitor cocktail (Sigma-Aldrich, St. Louis, MO). Precleared lysates containing 200 μg of whole lysate proteins diluted with lysis buffer were then mixed with 2 μg of protein G agarose conjugated acetyl lysine antibody (Cell Signaling) and incubated for 2 h. Samples were washed four times with lysis buffer and subjected to Western blot analysis.

### Determination of cell viabilities

The cytotoxicities of exogenous Aβ1–42 and endogenously released Aβwere assessed using a MTT [3-(4,5-dimethylthiazol-2-yl)-2,5-diphenyltetrazolium] assay. N2A and N2aSwe cells were seeded onto 12-well plates and cultured for 24 h before beginning the experiment. After one wash with PBS, cells were placed in a medium containing phenol red-free DMEM and 1% serum. Cells were pretreated with cilostazol when necessary for 2 h, and then 10 μM Aβ 1–42 was added and incubated for 24 h. Alternatively, cells were plated in Tet^+^ (1 μg/ml of tetracycline) or Tet^-^ condition containing vehicle or cilostazol with or without 3-methyladenine (3-MA, 2.5 mM). Wells containing medium without cells served as background controls, and cells cultured in Tet^+^ condition served as a positive control. The MTT assay was performed by adding 0.5 mg/ml of MTT and then incubating for 2 h at 37°C. The formazan salt generated by viable cells was dissolved in DMSO and absorbances were measured at 450 nm.

### Chemicals

Cilostazol [OPC-13013, 6-[4-(1-cyclohexyl-1*H*-tetrazol-5-yl) butoxy]-3,4-dihydro-2-(1*H*)-quinolinone] was donated by Otsuka Pharmaceutical Co. Ltd. (Tokushima, Japan) and dissolved in DMSO to prepare a 10 mM stock solution. Recombinant SIRT1, resveratrol, and bafilomycin A1 (Sigma-Aldrich) were dissolved in DMSO. Sirtinol (Calbiochem) was dissolved in DMSO (vehicle < 0.1% v/v on final volume). Aβ1–42 peptide (appearance, white powder; HPLC purity, >95%) was purchased from AnaSpec (catalog No.24236, run No. 77828; AnaSpec, Fremont, CA), dissolved in 1% NH_4_OH (basic buffer) as a stock solution at 1 mM and stored at -20°C. When diluted, the stock solution of Aβ1–42 was clear, seedless, and showed no sign of aggregation. KT5720 (a PKA inhibitor) was purchased from Enzo Life Sciences, and MTT, 3-methyladenine, bafilomycin A1, and tetracycline were from Sigma-Aldrich. TIMP-1 (Calbiochem) was dissolved in phosphate-buffered saline.

### Statistical Analyses

Results are expressed as means ± SDs. One-way analysis of variance followed by Tukey’s *post hoc* multiple comparisons was used to determine the significances of differences between vehicle and cilostazol treatment groups. Student’s *t*-test was used to determine the significance of difference between the mean of untreated cells and those treated with inhibitors. Statistical significance was accepted for *P* values of < 0.05.
